# Habitual Mouth Breathing

**Published:** 1881-12

**Authors:** 


### HABITUAL MOUTH BREATHING.

Many people sleep with the mouth open, and thus make this organ perform a duty which should be transacted by the nose. There are many objections to this. The air in passing through the channels of the nose, for instance, is raised to the temperature of the body before it reaches the larynx. Thus breathing, no matter how low the temperature may be, the sense of cold is never felt below the border of the soft palate. But when one breathes through the mouth on a cold day the sensation proceeds as far as the larynx, and an irritating cough may be caused. Then, again, in nose breathing the air is moistened by the natural secretions which cover the turbinated bones in a condition of health, and the short, bristly hairs at the opening of the nostrils act as a filter to arrest impurities and reduce the likelihood of laryngial, bronchial or pulmonary disease. Infants, athletes, savages and animals breathe through the nose; the ordinary civilized man employs the mouth to an unnecessary, and often to a very injurious, extent.

